# Assessment of Sentinel Lymph Node Biopsy vs Lymphadenectomy for Intermediate- and High-Grade Endometrial Cancer Staging

**DOI:** 10.1001/jamasurg.2020.5060

**Published:** 2020-11-11

**Authors:** Maria C. Cusimano, Danielle Vicus, Katherine Pulman, Manjula Maganti, Marcus Q. Bernardini, Genevieve Bouchard-Fortier, Stephane Laframboise, Taymaa May, Liat F. Hogen, Allan L. Covens, Lilian T. Gien, Rachel Kupets, Marjan Rouzbahman, Blaise A. Clarke, Jelena Mirkovic, Matthew Cesari, Gulisa Turashvili, Aysha Zia, Gabrielle E. V. Ene, Sarah E. Ferguson

**Affiliations:** 1 Department of Obstetrics and Gynaecology, University of Toronto, Toronto, Ontario, Canada; 2 Division of Gynecologic Oncology, Odette Cancer Centre, Sunnybrook Health Sciences Centre, Toronto, Ontario, Canada; 3 Gynecologic Oncology Program, Trillium Health Partners, Mississauga, Ontario, Canada; 4 Biostatistics Research Unit, University Health Network, Toronto, Ontario, Canada; 5 Division of Gynecologic Oncology, University Health Network/Sinai Health Systems, Toronto, Ontario, Canada; 6 Laboratory Medicine Program, University Health Network, Toronto, Ontario, Canada; 7 Department of Anatomic Pathology, Sunnybrook Health Sciences Centre, Toronto, Ontario, Canada; 8 Laboratory Medicine and Genetics Program, Trillium Health Partners, Mississauga, Ontario, Canada; 9 Department of Pathology and Laboratory Medicine, Sinai Health Systems, Toronto, Ontario, Canada

## Abstract

**Importance:**

Whether sentinel lymph node biopsy (SLNB) can replace lymphadenectomy for surgical staging in patients with high-grade endometrial cancer (EC) is unclear.

**Objective:**

To examine the diagnostic accuracy of, performance characteristics of, and morbidity associated with SLNB using indocyanine green in patients with intermediate- and high-grade EC.

**Design, Setting, and Participants:**

In this prospective, multicenter cohort study (Sentinel Lymph Node Biopsy vs Lymphadenectomy for Intermediate- and High-Grade Endometrial Cancer Staging [SENTOR] study), accrual occurred from July 1, 2015, to June 30, 2019, with early stoppage because of prespecified accuracy criteria. The study included patients with clinical stage I grade 2 endometrioid or high-grade EC scheduled to undergo laparoscopic or robotic hysterectomy with an intent to complete staging at 3 designated cancer centers in Toronto, Ontario, Canada.

**Exposures:**

All patients underwent SLNB followed by lymphadenectomy as the reference standard. Patients with grade 2 endometrioid EC underwent pelvic lymphadenectomy (PLND) alone, and patients with high-grade EC underwent PLND and para-aortic lymphadenectomy (PALND).

**Main Outcomes and Measures:**

The primary outcome was sensitivity of the SLNB algorithm. Secondary outcomes were additional measures of diagnostic accuracy, sentinel lymph node detection rates, and adverse events.

**Results:**

The study enrolled 156 patients (median age, 65.5 years; range, 40-86 years; median body mass index [calculated as weight in kilograms divided by height in meters squared], 27.5; range, 17.6-49.3), including 126 with high-grade EC. All patients underwent SLNB and PLND, and 101 patients (80%) with high-grade EC also underwent PALND. Sentinel lymph node detection rates were 97.4% per patient (95% CI, 93.6%-99.3%), 87.5% per hemipelvis (95% CI, 83.3%-91.0%), and 77.6% bilaterally (95% CI, 70.2%-83.8%). Of 27 patients (17%) with nodal metastases, 26 patients were correctly identified by the SLNB algorithm, yielding a sensitivity of 96% (95% CI, 81%-100%), a false-negative rate of 4% (95% CI, 0%-19%), and a negative predictive value of 99% (95% CI, 96%-100%). Only 1 patient (0.6%) was misclassified by the SLNB algorithm. Seven of 27 patients with node-positive cancer (26%) were identified outside traditional PLND boundaries or required immunohistochemistry for diagnosis.

**Conclusions and Relevance:**

In this prospective cohort study, SLNB had acceptable diagnostic accuracy for patients with high-grade EC at increased risk of nodal metastases and improved the detection of node-positive cases compared with lymphadenectomy. The findings suggest that SLNB is a viable option for the surgical staging of EC.

## Key Points

**Question:**

What is the diagnostic accuracy of sentinel lymph node biopsy (SLNB) compared with lymphadenectomy in women with intermediate- and high-grade endometrial cancer?

**Findings:**

In this cohort study of 156 patients with endometrial cancer (126 with high-grade histologic subtypes), SLNB had a sensitivity of 96% and a negative predictive value of 99% for the detection of nodal metastasis. A total of 26% of patients with node-positive cancer were identified outside lymphadenectomy boundaries or required immunohistochemistry for diagnosis.

**Meaning:**

In this study, SLNB had similar diagnostic accuracy and prognostic ability as lymphadenectomy in patients with high-grade endometrial cancer at greatest risk for nodal metastasis.

## Teaser

This cohort study examines the diagnostic accuracy, performance characteristics, and morbidity associated with sentinel lymph node biopsy in patients with intermediate- and high-grade endometrial cancer.

## Introduction

Endometrial cancer (EC) that has metastasized to surrounding lymph nodes is associated with a poor prognosis and requires administration of adjuvant therapy.^1^ Nodal metastases are traditionally identified on pelvic lymphadenectomy (PLND) and para-aortic lymphadenectomy (PALND), but 2 randomized clinical trials^2,3^ have suggested that lymph node resection independent of the effect of adjuvant therapy does not improve survival in patients with EC.

Sentinel lymph node biopsy (SLNB), or resection of only the first nodes receiving lymphatic drainage from the tumor site, has therefore been proposed as a less invasive strategy for nodal assessment.^4,5^ Theoretically, SLNB should reflect the status of the entire nodal basin and provide the pathologic information required to guide decisions on adjuvant therapy while avoiding the heightened risks of intraoperative injury, chronic lymphedema, and other complications associated with complete lymphadenectomy.^4,5,6^

Although SLNB has gained acceptance in the context of low-grade EC, its role in high-grade EC remains unclear. Only 13% of patients in the Sentinel Node and Endometrial Cancer (SENTI-ENDO) trial,^7^ 28% in the Determining the Sensitivity of Sentinel Lymph Nodes Identified With Robotic Fluorescence Imaging (FIRES) trial,^8^ and 49% in the Pelvic Sentinel Lymph Node Detection in High-Risk Endometrial Cancer (SHREC) trial^9^ had high-grade histologic subtypes. Of published studies that have evaluated SLNB predominantly in this patient population, most were retrospective and performed at a single center,^10,11,12^ did not perform PALND,^10,11,12^ or used technetium Tc 99m or blue dye rather than more contemporary tracers.^13^

Additional trials of SLNB followed by lymphadenectomy as the reference standard are needed to inform practice in EC. We therefore prospectively evaluated the performance characteristics of SLNB using indocyanine green (ICG) specifically in patients with clinical stage I disease with intermediate- and high-grade histologic subtypes. We hypothesized that SLNB would identify patients with nodal metastases with acceptable sensitivity.

## Methods

We conducted the Sentinel Lymph Node Biopsy vs Lymphadenectomy for Intermediate- and High-Grade Endometrial Cancer Staging (SENTOR) prospective, multicenter cohort study at 3 designated cancer centers in Toronto, Ontario, Canada.^14^ Provincial guidelines mandate that women with intermediate- and high-grade EC be referred to these centers for surgery.^15,16^ Research ethics boards at Princess Margaret Cancer Centre, Sunnybrook Health Sciences Centre, and Trillium Health Sciences approved this study. Written informed consent was obtained from participants. This study followed the Standards for Reporting of Diagnostic Accuracy (STARD) reporting guideline.

**Figure 1.**
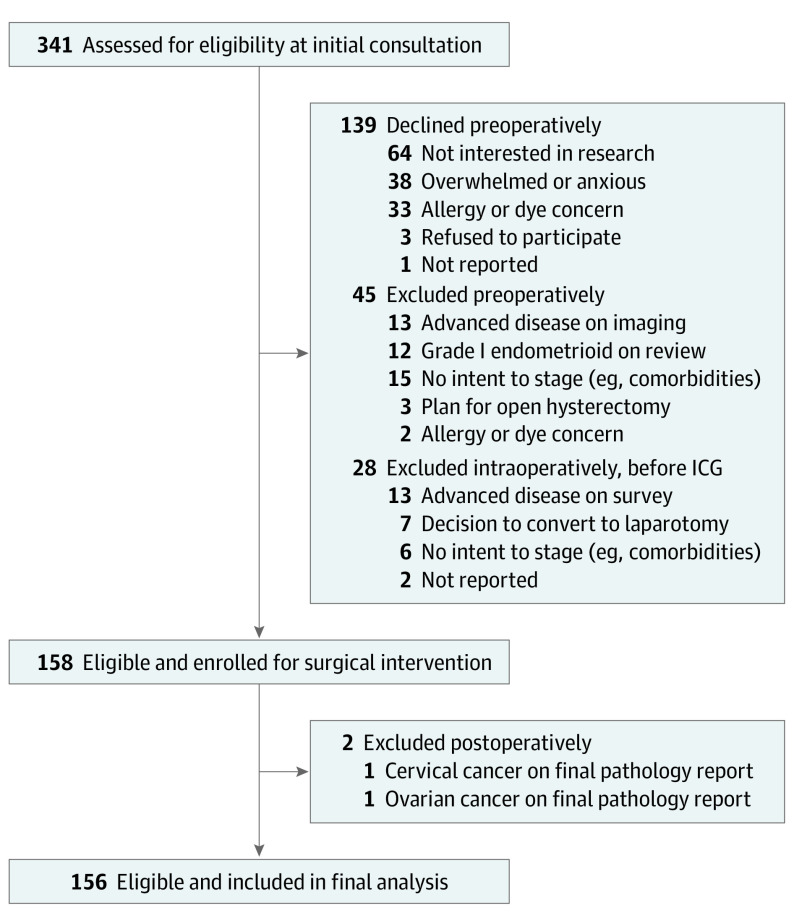
Flow Diagram of Included Patients ICG indicates indocyanine green.

Recruitment began July 1, 2015, and was stopped early according to prespecified accuracy criteria on June 30, 2019 (Figure 1). We enrolled consecutive patients (≥18 years of age) with clinical stage I grade 2 endometrioid or high-grade EC (grade 3 endometrioid, serous, carcinosarcoma, clear cell, undifferentiated or dedifferentiated, and mixed high grade) scheduled for laparoscopic or robotic primary hysterectomy with an intent to complete full staging. Potentially eligible patients were approached for written informed consent at the first surgical consultation and later excluded if pertinent information was noted on preoperative workup or initial intraoperative survey (before SLNB). We excluded patients with (1) grade 1 endometrioid, recurrent, or suspected advanced EC; (2) prior retroperitoneal surgery or abdominopelvic radiotherapy; (3) need for neoadjuvant therapy; (4) plans to omit lymphadenectomy based on surgical or anesthetic risk; (5) pregnancy; or (6) iodide allergy. Because of the low nodal event rates in patients with grade 2 endometrioid EC, protocols were amended in December 2017 to continue enrollment of patients with high-grade cancer only.

### Surgical Procedures

Operations were completed by 14 fellowship-trained gynecologic oncologists who had participated in a formal peer mentorship instruction program and validation study of SLNB technique led by the principal investigator (S.E.F.).^17^ Five surgeons (36%) had more than 10 years of postgraduate experience, 5 (36%) had 5 to 10 years, and 4 (28%) had 1 to 5 years.

During induction of anesthesia, one 25-mg vial of ICG (Akorn Inc) was reconstituted in 10 mL of sterile water (2.5 mg/mL) and drawn into a spinal needle. The cervix was injected at the 3- and 9-o'clock positions with 0.5 mL of IGC superficially (at 1- to 2-mm depth) and 0.5 mL of ICG deep (at 10-mm depth) for a total dose of 2 mL of ICG. Laparoscopy was subsequently initiated with an abdominal survey to confirm feasibility of lymphadenectomy and note final reasons for exclusion (eg, intraperitoneal metastases) before proceeding to SLNB.

Patients then underwent a standard algorithm for SLNB.^18^ In the first step, each hemipelvis was assessed for successful mapping of a sentinel lymph node. Surgeons entered the retroperitoneum over the psoas muscle, developed the pararectal and paravesical spaces carefully to preserve afferent lymphatic channels, and identified sentinel lymph nodes using the Pinpoint Endoscopic Fluorescence Imaging System (Novadaq Technologies). All green nodes and nongreen nodes with green afferent lymphatic channels were deemed sentinel lymph nodes. In the second step, sentinel lymph nodes were resected from mapped hemipelves, and locations were noted by the surgeon on standardized intraoperative data collection forms. In the final step, side-specific lymphadenectomy was performed on nonmapped hemipelves, which entailed side-specific PLND alone (internal iliac, external iliac, and obturator lymph nodes) for patients with grade 2 endometrioid EC or side-specific PLND and PALND (aortic bifurcation to inferior mesenteric artery) for patients with high-grade cancer (eMethods in the Supplement).

Patients then underwent the reference standard of lymphadenectomy; grade 2 endometrioid EC required bilateral PLND, and high-grade EC required bilateral PLND and PALND (boundaries as above). Patients with grade 2 endometrioid EC underwent PALND only when a sentinel lymph node mapped to the para-aortic region or when the surgeon deemed it necessary. After the SLNB algorithm was complete, patients underwent total hysterectomy, bilateral salpingo-oophorectomy, and omental biopsy.

### Histopathologic Procedures

Sentinel lymph nodes were handled using a standardized ultrastaging protocol at all centers, with nodes cut at 2-mm intervals perpendicular to the long axis in a bread-loaf fashion. The first section was processed immediately as a frozen section; this processing was performed for research purposes only, did not impact surgical protocols or decision-making, and is not discussed further here. Sentinel lymph nodes were then fixed in formalin and embedded in paraffin. Two sections were obtained from each paraffin block at 50 μM apart and stained with hematoxylin-eosin; a third section was taken directly after the first section and evaluated by immunohistochemistry for pan-cytokeratin AE1/AE3 (Dako). Nonsentinel lymph nodes were bisected parallel to the long axis and stained with hematoxylin-eosin. Pathologists were aware of the sentinel lymph node status before assessing nonsentinel lymph nodes. Nodal metastases were classified as (1) isolated tumor cells (ITCs) (single cells or clusters ≤0.2 mm in largest dimension), (2) micrometastases (tumor deposits 0.2-2 mm), or (3) macrometastases (tumor deposits >2 mm). Patients with ITCs were considered to have node-positive disease. All specimens were read by a gynecologic pathologist (M.R., B.A.C., J.M., and G.T.).

### Adverse Events

Intraoperative events, defined as injuries or undesired events occurring between skin incision and closure, were categorized according to timing in surgery and organ system affected (eTable 1 in the Supplement). Postoperative events, defined as any deviation from a normal postsurgical course within 30 days postoperatively, were categorized according to the Clavien-Dindo surgical grading system (eTables 2 and 3 in the Supplement).^19,20^

### Outcomes

The primary end point was the sensitivity of the SLNB algorithm in detecting metastatic disease. Sensitivity was defined as the proportion of patients with node-positive disease identified by the SLNB algorithm (ie, positive node on sentinel lymph node specimens for mapped hemipelves or lymphadenectomy specimens for nonmapped hemipelves).^18^ This outcome was selected because it would be most clinically relevant to individual patients and decisions on whether to administer adjuvant therapy. We also determined the false-negative rate (FNR), defined as the proportion of patients with node-positive disease not identified by the SLNB algorithm, and the negative predictive value (NPV), defined as the proportion of patients considered negative for metastatic disease according to the SLNB algorithm who truly had node-negative disease.

Secondary end points were measures of diagnostic accuracy for the sentinel lymph node specimen. Sensitivity was defined as the proportion of node-positive hemipelves identified by the sentinel lymph node, FNR was defined as the proportion of node-positive hemipelves in which the sentinel lymph node was negative, and NPV was defined as the proportion of hemipelves with a negative sentinel lymph node that was truly node negative. Other secondary end points were the patient-specific detection rate, defined as the proportion of patients in whom a sentinel lymph node mapped; side-specific detection rate, defined as the proportion of hemipelves in which a sentinel lymph node mapped; and bilateral detection rate, defined as the proportion of patients in whom sentinel lymph nodes mapped bilaterally.

### Statistical Analysis

All enrolled patients were included in the analyses for diagnostic accuracy of the SLNB algorithm (primary end point) and detection rates (secondary end point). Hemipelves with at least 1 mapped sentinel lymph node and a corresponding lymphadenectomy were included in the analyses for diagnostic accuracy of the sentinel lymph node specimen (secondary end point). Sentinel lymph node pathology was compared with nonsentinel lymph node pathology within the same patient or hemipelvis depending on the end point.

Sample size was determined based on our primary outcome of algorithm-specific sensitivity. We used a Fleming 2-stage design to test the null hypothesis that the sensitivity was 80% against a 1-sided alternative that the sensitivity was 93%.^21,22^ Values were selected from a meta-analysis of 26 studies including 1101 SLNB procedures.^23^ Assuming an estimated 20% node-positivity rate, we required 46 patients with node-positive disease from an estimated 230 patients recruited in 2 stages to test this hypothesis^22^ (eMethods in the Supplement). In the first stage, 25 patients with node-positive disease were enrolled; the study would stop early for unacceptable accuracy if 20 patients or fewer were identified by the algorithm or for acceptable accuracy if 24 patients or more were identified by the algorithm.^22^ This target was met in June 2019. Accrual would have otherwise continued in a second stage to the total of 46 patients with node-positive disease, and the null hypothesis would have been rejected if 42 or more were accurately identified by the algorithm. This design yields a type I error rate of .05 and power of 0.8 when the true sensitivity was 93%.

Descriptive statistics were used to characterize the study cohort. We calculated sensitivity, NPV, FNR, and detection rate using proportions and generated 95% CIs based on the exact (Clopper-Pearson) method. Analyses were performed using SAS statistical software, version 9.4 (SAS Institute Inc).

## Results

**Figure 2.**
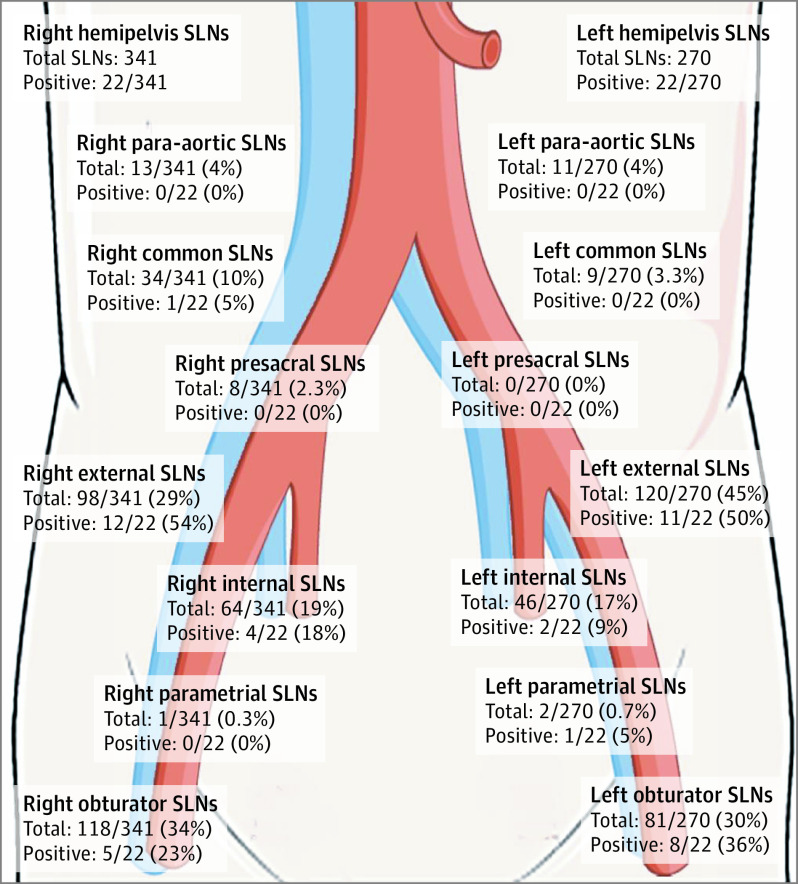
Anatomical Location of Sentinel Lymph Nodes (SLNs) Figure adapted with permission from Servier Medical Art (http://www.servier.com).

During the study period, 156 patients (median age, 65.5 years; range, 40-86 years; median body mass index [calculated as weight in kilograms divided by height in meters squared], 27.5; range, 17.6-49.3) were enrolled; 30 (19.2%) had grade 2 endometrioid EC, and 126 (81%) had high-grade EC (Table 1). All patients underwent the SLNB algorithm, with a detection rate of 97.4% per patient (95% CI, 93.6%-99.3%), 87.5% per hemipelvis (95% CI, 83.3%-91.0%), and 77.6% bilaterally (95% CI, 70.2%-83.8%) (Table 2). Surgeons resected a median of 3 (interquartile range [IQR], 2-5) sentinel lymph nodes per patient and a total of 611 sentinel lymph nodes overall (right, 341; left, 270) (Figure 2).

**Table 1.** Baseline Characteristics of Enrolled Patients

Characteristic	Enrolled patients (N = 156)^a^
**Clinical findings**	
Age, median (IQR) [range], y	65.5 (61.0-70.0) [40-86]
BMI, median (IQR) [range]	27.5 (24.3-32.2) [17.6-49.3]
Menopausal status	
Premenopausal	15 (9.6)
Postmenopausal	141 (90.4)
Hypertension	
Yes	75 (48.1)
No	81 (51.9)
Diabetes	
Yes	31 (19.9)
No	125 (80.1)
Hysterectomy type	
Simple	155 (99.4)
Radical	1 (0.6)
Surgical approach	
Robotic	26 (16.7)
Laparoscopic	130 (83.3)
**Pathology**	
Histologic subtype	
Grade 2 endometrioid	30 (19.2)
Grade 3 endometrioid	35 (22.5)
Serous	52 (33.4)
Clear cell	3 (1.9)
Carcinosarcoma	17 (10.9)
Undifferentiated or dedifferentiated	5 (3.2)
Mixed	13 (8.3)
High-grade NOS	1 (0.6)

Abbreviations: BMI, body mass index (calculated as weight in kilograms divided by height in meters squared); IQR, interquartile range; NOS, not otherwise specified.

^a^ Data are presented as number (percentage) of patients unless otherwise indicated.

**Table 2.** Postoperative Characteristics of Enrolled Patients

Characteristic	Enrolled patients (N = 156)^a^
**Surgical**	
Sentinel lymph node detection	
Any	152 (97.4)
Bilateral	121 (77.6)
Pelvic lymphadenectomy	156 (100)
Para-aortic lymphadenectomy^b^	101 (80.2)
Lymph nodes removed, median (IQR), No.	
Sentinel	3 (2-5)
Pelvic	16 (12-20)
Para-aortic	5 (3-9)
**Pathology**	
Lymph node metastases	
Yes	27 (17.3)
No	129 (82.7)
Lymphovascular space invasion	
No residual tumor	11 (7.1)
Yes	60 (38.4)
No	85 (54.5)
Myometrial invasion	
No residual tumor	11 (7.1)
No invasion	29 (18.6)
<50%	75 (48.1)
≥50%	41 (26.2)
FIGO stage	
IA	93 (59.6)
IB	20 (12.8)
II	12 (7.7)
IIIA	3 (1.9)
IIIC1	19 (12.2)
IIIC2	8 (5.2)
IV	1 (0.6)

Abbreviations: FIGO, International Federation of Gynecology and Obstetrics; IQR, interquartile range.

^a^ Data are presented as number (percentage) of patients unless otherwise indicated.

^b^ The denominator was 126.

Patients subsequently underwent the reference standard; 156 (100%) underwent PLND, and 101 patients (80.2%) with high-grade EC also underwent PALND (Table 2). Surgeons resected a median of 16 (IQR, 12-20) pelvic and 5 (IQR, 3-9) para-aortic lymph nodes. Removal of 10 or more pelvic lymph nodes was performed in 134 patients (85%).^24^

Twenty-seven patients (17%) had metastatic disease in their sentinel lymph node or lymphadenectomy specimens (Table 2); 24 had high-grade EC (grade 3 endometrioid, 3; serous, 15; carcinosarcoma, 2; dedifferentiated, 1; and mixed high-grade, 3), and only 3 had grade 2 endometrioid EC. This total included the 25 patients who triggered initial stoppage of the study in June 2019 and 2 patients who had undergone surgery before that point and whose pathology reports became available only after interim analyses were complete.

Our primary analysis included all 156 patients. Twenty-six of 27 patients with node-positive disease were correctly identified by the SLNB algorithm, yielding a sensitivity of 96.3% (26 of 27 patients; 95% CI, 81.0%-99.9%), an FNR of 3.7% (1 of 27; 95% CI, 0.1%-19.0%), and an NPV of 99.2% (129 of 130; 95% CI, 95.8%-99.9%) (Table 3). Of the 26 patients with node-positive disease identified by the SLNB algorithm, 24 were diagnosed based on sentinel lymph nodes from mapped hemipelves and 2 were diagnosed based on side-specific PLND or PALND specimens from nonmapped hemipelves; these 2 patients had high-grade EC (serous and carcinosarcoma) and a single positive pelvic or para-aortic lymph node. Only 1 patient in the total cohort (0.6%) was misclassified by SLNB and deemed to have a false-negative result. This patient had dedifferentiated histologic findings, lymphovascular space invasion, and greater than 50% myometrial invasion on the final pathology report; results of bilateral mapped sentinel lymph nodes were negative, but 2 right PLND and 2 right PALND nodes tested positive.

**Table 3.** Primary and Secondary Outcomes

Result	LND positive, No.	LND negative, No.	Total, No.
**SLNB algorithm (patient specific)**			
SLNB positive	26	0	26
SLNB negative	1	129	130
Total	27	129	156
**SLN (hemipelvis specific)**			
SLN positive	32	0	32
SLN negative	2	236	238
Total	34	236	270

Abbreviations: LND, lymphadenectomy; SLN, sentinel lymph node; SLNB, sentinel lymph node biopsy.

Our secondary analysis included 270 hemipelves in which both a sentinel lymph node was mapped and lymphadenectomy was performed. Thirty-two of 34 node-positive hemipelves were correctly identified by the sentinel lymph node, yielding a sensitivity of 94.1% (32 of 34 cases; 95% CI, 80.3%-99.3%), an FNR of 5.9% (2 of 34; 95% CI, 0.7%-19.7%), and an NPV of 99.1% (236 of 238; 95% CI, 97.0%-99.9%) (Table 3).

Of the 27 patients with node-positive disease, 14 (52%) had metastases in the sentinel lymph node specimen only, 10 (37%) had metastases in the sentinel lymph node and lymphadenectomy specimens, and 3 (11%) had metastases in the lymphadenectomy specimen only (2 with unilateral mapping and 1 with bilateral mapping but false-negative sentinel lymph node specimens). Two patients with node-positive disease (7.5%) had a single metastatic sentinel lymph node mapped outside traditional PLND boundaries (1 parametrial and 1 common iliac), and 5 patients with node-positive disease (18.5%) required immunohistochemistry for diagnosis (total of 7 patients with node-positive disease [26%]). Of the 14 patients with metastases in the sentinel lymph node only, all had micrometastases or ITCs.

Adverse events are reported in eTables 1 through 4 in the Supplement. Five patients (3%) experienced an intraoperative adverse event; none were during SLNB, and 2 were during PLND or PALND. Forty-one patients (26%) experienced at least 1 postoperative adverse event within 30 days of surgery, but 36 of 41 (88%) were minor (grades 1-2).

## Discussion

The SENTOR study was powered to prospectively evaluate the diagnostic accuracy of SLNB using ICG in patients with intermediate- and high-grade EC. More than 96% of patients with node-positive disease were correctly identified by an SLNB algorithm,^1,18^ and 99% of patients with negative sentinel lymph nodes had node-negative disease. These measures are comparable to those observed for breast cancer^25^ and melanoma,^26^ for which SLNB has become the standard of care, and suggest that endometrial SLNB has the performance characteristics required to be trialed as a replacement for lymphadenectomy.

The SENTOR study adds to previous work^8,9,13^ by being applicable to patients with high-grade EC; more than 80% of the total cohort, 89% of all patients with node-positive disease identified, and 24 of the minimum required 25 patients with node-positive disease (96%) had high-grade EC. Studies by Rossi et al,^8^ Persson et al,^9^ and Soliman et al^13^ found similar sensitivities (96%-98%) and negative predictive values (99%) but were focused on patients who had grade 1 to 2 endometrioid EC (254 of 356 [71%]),^8^ who had a heterogeneous mix of high-risk features,^9^ or who had received blue dye or technetium Tc 99m (39 of 101 [40%]).^13^ We found that SLNB had acceptable diagnostic accuracy with a more contemporary tracer in a cohort largely composed of patients with high-grade disease.

The SENTOR study also suggests that SLNB may improve the detection of nodal metastases in ways not captured by traditional calculations of diagnostic accuracy. Fourteen patients with node-positive disease (52%) had metastatic disease in sentinel lymph nodes only, and 7 cases (26%) were found outside lymphadenectomy boundaries or required immunohistochemistry for diagnosis. These patients would not have been identified by PLND or PALND alone. In the FIRES trial, sentinel lymph nodes contained metastatic disease more often than nonsentinel lymph nodes (58 of 1098 [5%] vs 63 of 5416 [1%], *P* < .001), and 54% of patients with positive sentinel lymph nodes had low-volume metastases that would have been missed without ultrastaging.^8^ Studies^27,28,29,30,31^ in breast and gynecologic cancers suggest that SLNB increases detection of micrometastases and ITCs by 4% to 25%. Although such small-volume metastases may have little prognostic significance regardless of adjuvant therapy in patients with low-grade EC,^32,33^ their association with oncologic outcomes in patients with high-grade EC remains unclear. The Randomized Trial of Radiation Therapy With or Without Chemotherapy for Endometrial Cancer (PORTEC-3) trial further demonstrates that patients with high-grade EC and lymph node metastases derive a survival benefit from adjuvant chemotherapy.^34^ As a result, it is crucial that we continue to identify patients with high-grade EC with small-volume metastases, and this appears to be achieved most effectively with SLNB.

On the basis of these data and existing literature, SLNB could potentially replace lymphadenectomy for the surgical staging of both low- and high-grade EC. Two randomized clinical trials^2,3^ found that lymph node resection does not improve survival among patients with EC; rather, the accurate identification of nodal metastases offers prognostic information that may direct administration of adjuvant therapy to patients who will benefit. Our study suggests that SLNB has comparable, if not improved, diagnostic accuracy and prognostic ability compared with lymphadenectomy in patients with high-grade EC and should be considered for the surgical staging of apparent clinical stage I EC with no evidence of extrauterine disease on imaging or intraoperative survey.

If SLNB is to be adopted, surgeons must strictly follow an SLNB algorithm that incorporates both side-specific PLND and PALND for nonmapped hemipelves in patients with high-grade EC.^18^ Sentinel lymph nodes may not map when infiltrated with tumor or when lymphatic drainage is altered,^18^ and this may be particularly common in patients with high-grade EC at increased risk of nodal metastasis. Two of 27 patients with node-positive disease (7.5%) with unilateral mapping in our study would have been missed without a side-specific lymphadenectomy that included PALND.^18^ We also propose that initial adoption of SLNB occur alongside continued performance of PLND and PALND so that centers can document proficiency. Our surgeons participated in a validation study^17^ of cervical SLNB before initiating the SENTOR study for endometrial SLNB; we accordingly achieved a bilateral detection rate of 78%.^17^ Comparable prospective studies^8,13^ with surgeons for whom SLNB was a novel technique have reported bilateral detection rates of 52% to 58%.

### Strengths and Limitations

This study has strengths. The SENTOR study is an important addition to the literature because of its rigorous prospective design, use of both PLND and PALND as the reference standard, and statistical power to assess the diagnostic accuracy of SLNB, specifically in patients with intermediate- and high-grade EC. This study also has limitations. Our estimates of diagnostic accuracy may not be generalizable to less experienced surgeons and centers, to SLNB with different types of tracers, to patients who would not typically participate in surgical trials, or to patients in whom PLND or PALND may not be feasible. We also cannot comment on the survival, recurrence, and morbidity associated with SLNB alone. Future randomized clinical trials may consider comparing these outcomes between SLNB alone and no lymph node assessment.

## Conclusions

In this study, SLNB had acceptable diagnostic accuracy compared with lymphadenectomy for the detection of nodal metastatic disease in high-grade EC. On the basis of this study and the existing literature, SLNB appears to be a viable option for the surgical staging of both low- and high-grade EC.

## Notes

Supplement. **eMethods.** Additional Description of Study Methods **eTable 1.** Intraoperative Adverse Event Log **eTable 2.** Clavien-Dindo Surgical Grading System **eTable 3.** Postoperative Adverse Event Log **eTable 4.** Summary of Intraoperative and 30-Day Postoperative Adverse Event
