# Supplementary material for: Assessment of Sentinel Lymph Node Biopsy vs Lymphadenectomy for Intermediate- and High-Grade Endometrial Cancer Staging
Source: JAMA Surg. 2020 Nov 11;156(2):1–8. doi: 10.1001/jamasurg.2020.5060 (PMC7658802; doi:10.1001/jamasurg.2020.5060)
Supplement: Supplement. — eMethods. Additional Description of Study Methods eTable 1. Intraoperative Adverse Event Log eTable 2. Clavien-Dindo Surgical Grading System eTable 3. Postoperative Adverse Event Log eTable 4. Summary of Intraoperative and 30-Day Postoperative Adverse Event [file jamasurg-e205060-s001.pdf]

Supplementary Online Content

Cusimano MC, Vicus D, Pulman K, et al. Assessment of sentinel lymph node bipsy vs lymphadenectomy for intermediate- and high-grade endometrial cancer staging. *JAMA Surg.* Published online November 11, 2020. doi:10.1001/jamasurg.2020.5060

eMethods. Additional Description of Study Methods

eTable 1. Intraoperative Adverse Event Log

eTable 2. Clavien-Dindo Surgical Grading System

eTable 3. Postoperative Adverse Event Log

eTable 4. Summary of Intraoperative and 30-Day Postoperative Adverse Events

This supplementary material has been provided by the authors to give readers additional information about their work.

© 2020 Cusimano MC et al. *JAM Surgery*.

eTable 1. Intraoperative adverse events

ID	Timing	Type	Description
ABL-117-MD	Entry	Genitourinary	Bladder injury from trocar insertion. Repaired intraoperatively and cystoscopy confirmed intact bladder. Foley catheter placed and removed POD#10.
ABL-121-HS	Entry	Gastrointestinal	Concern of possible serosal gastric injury from trocar insertion. Single suture placed. Nasogastric tube kept in place during immediate postoperative period.
ABL-124-BS	Intraoperative NOS	Gastrointestinal	Serosal injury to sigmoid colon during manipulation of bowel. Single suture placed to close the defect.
SB-022-AM	Para-aortic lymphadenectomy	Vascular	Injury to IVC when right para-aortic nodes lifted off.
SB-070-YDP	Para-aortic lymphadenectomy	Vascular	Small perforator bleeder off IVC. Controlled with clips.

Abbreviations: NOS (not otherwise specified); POD (postoperative day); IVC (inferior vena cava)

© 2020 Cusimano MC et al. *JAM Surgery*.

eTable 2. Clavien-Dindo surgical grading system. Major (grade 3-5) and minor (grade 1-2) postoperative events were reported to the principal investigator within 24 hours and 30 days of detection respectively.

**Grade**	**Definition**
Grade 1	Any deviation from the normal postoperative course without the need for pharmacological treatment or surgical, endoscopic and radiological interventions.<br>Allowed therapeutic regimens are: drugs as anti-emetics, antipyretics, analgesics, diuretics and electrolytes and physiotherapy. This grade also includes wound infections opened at the bedside.
Grade 2	Requiring pharmacological treatment with drugs other than such allowed for grade I complications.<br>Blood transfusions and total parenteral nutrition are also included.
Grade 3	Requiring surgical, endoscopic or radiological intervention
Grade 3a	Intervention not under general anesthesia
Grade 3b	Intervention under general anesthesia
Grade 4	Life-threatening complication (including CNS complications)* requiring IC/ICU-management
Grade 4a	Single organ dysfunction (including dialysis)
Grade 4b	Multi-organ dysfunction
Grade 5	Death of patient

Abbreviations: CNS (central nervous system); IC (intermediate care); ICU (intensive care unit)
*Central nervous system complications include brain hemorrhage, ischemic stroke, subarachnoid bleeding, but exclude transient ischemic attacks (TIA)

© 2020 Cusimano MC et al. *JAM Surgery*.

eTable 3. Postoperative adverse events

ID	Type	Grade	Description
ABL-025-HHE	Gastrointestinal	1	Hernia. Asymptomatic hernia at RLQ port site. No intervention required.
ABL-028-MGS	SSI Organ Space	2	Pelvic abscess. ER visits with fever and foul-smelling vaginal discharge, WBC count 17, CT consistent with pelvic abscess. Treated with oral antibiotics.
ABL-030-JR	Genitourinary	2	UTI. Treated with oral antibiotics.
ABL-041-PM	Neurological	1	Left leg numbness. No intervention required.
ABL-042-MEP	SSI Superficial Incisional	2	LLQ port site infection. Treated with oral antibiotics.
ABL-044-LC	Cardiorespiratory	1	Shortness of breath NYD. PE ruled out. Symptoms resolved. No intervention required.
ABL-050-BW	Neurological	1	Right leg numbness. No intervention required.
ABL-051-CD	Pain	1	Increased postoperative pain. Emergency department visit requiring prescription of opioids.
ABL-051-CD	Pain	1	Rash NYD. Emergency department visit requiring prescription of antihistamine.
ABL-054-AP	Genitourinary	1	Urinary retention. High post-void residuals postoperatively requiring placement of Foley catheter from POD#1 to POD#2.
ABL-056-LG	SSI Organ Space	1	Vaginal cuff seroma. Resolved spontaneously. No intervention required.
ABL-062-RK	Cardiorespiratory	1	Shortness of breath NYD. Symptoms resolved. No intervention required.
ABL-070-FP	Neurological	1	Left leg weakness. No intervention required.
ABL-073-KD	Genitourinary	1	Vaginal bleeding. No intervention required.
ABL-078-MAP	Genitourinary	1	Vaginal bleeding. No intervention required.

© 2020 Cusimano MC et al. *JAM Surgery*.

ABL-079-IB	Genitourinary	1	Vaginal bleeding. No intervention required.
ABL-082-JS	Genitourinary	1	Urinary retention. Failed trial of void requiring prolonged admission and placement of Foley catheter.
ABL-083-BG	SSI Deep Incisional	2	Small wound dehiscence. Required prescription of oral antibiotics but no further intervention.
ABL-098-FT	Genitourinary	1	Vaginal discharge NYD. Resolved spontaneously. No further intervention required.
ABL-105-LB	Neurological	2	Herpes zoster (forehead). Prescribed antivirals and referred to Ophthalmology.
ABL-107-SM	SSI Deep Incisional	2	Serous wound drainage. Prescribed oral antibiotics.
ABL-115-LLY	Genitourinary	1	Vaginal bleeding. Silver nitrate to vaginal vault.
ABL-126-MAM	Pain	1	Left hip pain. DVT ruled out. Spontaneously resolved. No further intervention required.
ABL-128-MEM	Neurological	1	Right leg weakness. Referral to Neuro-Oncology. Investigations suggested right obturator neuropathy. No further intervention required.
ABL-137-SBC	Neurological	4A	CNS complication. Fluctuating consciousness in PACU with GSC=3 upon extubation. Admitted to ICU.
ABL-137-SBC	Neurological	1	Femoral nerve neuropathy. Noted on return to ward. No further intervention required.
ABL-144-PK	Genitourinary	1	Urinary retention. Insertion of Foley catheter from POD#1 to POD#2.
ABL-153-HM	SSI Superficial Incisional	2	Suspected superficial port site infection. Topical agent prescribed by family physician. No signs of infection seen on follow-up with Gynecologic Oncology.
ABL-157-RF	SSI Deep Incisional	2	Wound infection. Managed by opening & packing, and prescribing oral antibiotics.
ABL-157-RF	Neurological	1	Left leg weakness. No further intervention required.

© 2020 Cusimano MC et al. *JAM Surgery*.

ABL-157-RF	Vascular	1	Lymphocyst. No further intervention required.
ABL-159-LMS	Gastrointestinal	3B	Incarcerated hernia and small bowel obstruction. Urgent surgery for reduction of hernia with bowel resection, and postoperative ventral hernia repair.
ABL-166-GNB	Genitourinary	2	UTI. Treated with oral antibiotics.
SB-020-AA	Genitourinary	1	Vaginal bleeding. No further intervention required.
SB-026-AA	Gastrointestinal.	3A	Small bowel obstruction. Managed with admission and placement of nasogastric tube.
SB-030-MC	Genitourinary	1	Urinary retention. High post-void residuals requiring re-insertion of Foley catheter.
SB-041-PL	Genitourinary	2	UTI. Treated with oral antibiotics.
SB-041-PL	Vascular	1	Left leg lymphedema. DVT ruled out. Spontaneously resolved. No further intervention required.
SB-048-MT	Neurological	6	Increased resting tremor due to anesthetic. Spontaneously resolved. No further intervention required.
SB-067-WF	Neurological	1	Left leg numbness. No further intervention required.
SB-071-ZP	SSI Organ Space	3A	Multiple abdominal abscesses. Required drain insertion and IV antibiotics in hospital.
SB-071-ZP	Gastrointestinal	3A	Ileus secondary to multiple abdominal abscesses. Required drain insertion and IV antibiotics in hospital.
SB-071-ZP	Cardiorespiratory	1	Congestive heart failure. Managed with diuretics and rehabilitation.
SB-071-ZP	Genitourinary	1	Urinary retention. Required re-insertion of Foley catheter while in hospital.

© 2020 Cusimano MC et al. *JAM Surgery*.

SB-081-SP	Gastrointestinal	3B	Incarcerated femoral hernia with associated SBO. Required urgent surgical repair.
SB-084-SC	SSI Superficial Incisional	1	Serous drainage from umbilical port site. No further intervention required.
SB-089-TC	Pain	1	Increased abdominal pain. Investigations demonstrated pelvic and para-aortic adenopathy but no other etiology. Prescribed opioids.
CVH-1-SLN	Vascular	2	DVT. Prescribed anticoagulants.
CVH-6-SLN	Genitourinary	2	UTI. Prescribed oral antibiotics.
CVH-6-SLN	Neurological	1	Dizziness NYD. Spontaneously resolved. No further intervention required.

© 2020 Cusimano MC et al. *JAM Surgery*.

eMethods. Additional description of study methods

1) Sentinel Lymph Node Biopsy in Endometrial Cancer

The adequacy or quality of SLNB is ensured through an evaluation of detection or mapping (i.e. successful identification of a sentinel lymph node by dye accumulation) and diagnostic accuracy (i.e. performance of SLNB in diagnosing nodal metastases relative to complete lymphadenectomy). In malignancies such as breast cancer, with unilateral lymphatic drainage, SLNB performance characteristics are calculated at the level of the patient only. However, the uterus and cervix are midline structures with bilateral lymphatic drainage, so the sentinel lymph node status on one side of the pelvis does not predict the presence or absence of metastasis on the contralateral side. It has therefore been proposed that SLNB performance characteristics should be reported at the level of the patient and at the level of the hemipelvis or the side of dissection (i.e. side-specific).

2) Sample Size Calculations

We used a Fleming two-stage design, in line with numerous other studies in this field[1,2], to have the potential option of stopping early based on sensitivity. We aimed to determine if sentinel lymph node biopsy had sufficient diagnostic accuracy (i.e. sensitivity) to warrant further investigation. The Fleming two-stage design permits early stopping of the study if interim results are extreme – in our case, either demonstrating very high or very low sensitivity. Although multi-stage designs are typically used for phase II clinical trials, they can also be applied to studies in which diagnostic accuracy is the primary focus[3-6].

We tested the test the null hypothesis that the sensitivity was 80% (P0) against a one-sided alternative that the SN was 93% (P1). P0 is defined as the largest value for sensitivity which clearly implies that SLNB is an unacceptable intervention; in our case, if the sensitivity was $\leq$80%, we would have determined that SLNB had unacceptably low diagnostic accuracy and need not be studied further. P1 is defined as the smallest value for sensitivity which clearly implies that SLNB is an acceptable intervention; in our case, if the sensitivity was $\geq$93%, we would have determined that SLNB had acceptably high sensitivity and should be recommended. We selected values for P0 and P1 based on existing systematic reviews and consensus among gynecologic oncologists as to the minimum sensitivity needed for SLNB to be adopted[7].

1) Rossi EC, Kowalski LD, Scalici J, et al. A comparison of sentinel lymph node biopsy to lymphadenectomy for endometrial cancer staging (FIRES trial): a multicentre, prospective, cohort study. *Lancet Oncol.* 2017;18(3):384-392.

2) Persson J, Salehi S, Bollino M, Lonnerfors C, Falconer H, Geppert B. Pelvic Sentinel lymph node detection in High-Risk Endometrial Cancer (SHREC-trial)-the final step towards a paradigm shift in surgical staging. *Eur J Cancer.* 2019;116:77-85.

3) Zapf A, Stark M, Gerke O, et al. Adaptive trial designs in diagnostic accuracy research. Stat Med. 2020; 39(5):591-601.

4) Mazumdar M. Group sequential design for comparative diagnostic accuracy studies: implications and guidelines for practitioners. *Med Decis Making*. 2004;24(5):525-33..

5) Mazumdar M, Liu A. Group sequential design for comparative diagnostic accuracy studies. *Stat Med*. 2003;22(5):727-39.

6) Pepe MS, Feng Z, Longton G, et al. Conditional estimation of sensitivity and specificity from a phase 2 biomarker study allowing early termination for futility. *Stat Med*. 2009;28(5):762-79.

7) Kang S, Yoo HJ, Hwang JH, Lim MC, Seo SS, Park SY. Sentinel lymph node biopsy in endometrial cancer: meta-analysis of 26 studies. *Gynecol Oncol.* 2011;123(3):522-527.

© 2020 Cusimano MC et al. *JAM Surgery*.

eTable 4. Summary of intraoperative and 30-day postoperative adverse events

**Characteristic**	**Enrolled Patients (N=156)**
**Intraoperative Adverse Events**	
Events experienced, *No. (%)*	N=156
0	151 (96.8)
1	5 (3.2)
2+	0 (0.0)
Type of event, *No. (%)*	N=5
Vascular	2 (40.0)
Genitourinary	1 (20.0)
Gastrointestinal	2 (40.0)
Timing of event, *No. (%)*	N=5
Entry or closure	2 (40.0)
Pelvic or para-aortic lymphadenectomy	2 (40.0)
Not otherwise specified	1 (20.0)
**Postoperative Adverse Events**	
Events experienced, *No. (%)*	N=156
0	115 (73.7)
1	34 (21.8)
2+	7 (4.5)
Highest grade event, *No. (%)*	N=41
1	24 (58.5)
2	12 (29.3)
3a	2 (4.9)
3b	2 (4.9)
4a	1 (2.4)
4b	0 (0.0)
5	0 (0.0)
Type by highest grade event, *No. (%)*	N=41
Vascular	1 (2.4)
Neurological	8 (19.5)
Genitourinary	13 (31.7)
Gastrointestinal	4 (9.8)
Cardiorespiratory	2 (4.9)
Superficial Incisional	4 (9.8)
Deep Incisional	3 (7.3)
Organ Space	3 (7.3)
Pain	3 (7.3)

Abbreviations: No. (number)

© 2020 Cusimano MC et al. *JAM Surgery*.
